# The relationship of diabetic retinopathy severity scales with frequency and surface area of diabetic retinopathy lesions

**DOI:** 10.1007/s00417-023-06145-7

**Published:** 2023-07-01

**Authors:** Houri Esmaeilkhanian, Henry Liu, Sohaib Fasih-Ahmed, Ramya Gnanaraj, Aditya Verma, Deniz Oncel, Ye He, Muneeswar Gupta Nittala, Yamini Attiku, Shin Kadomoto, Giulia Corradetti, Swetha Bindu Velaga, Irena Tsui, Pradeep Prasad, Xiaorong Li, Xiao Li, Shangjun Collier Jiang, Netan Choudhry, Chaitra Jayadev, SriniVas Sadda

**Affiliations:** 1 Doheny Image Reading and Research Laboratory, Doheny Eye Institute, David Geffen School of Medicine, 150 N. Orange Grove Blvd, Suite 232, Pasadena, CA 91103 USA; 2 Department of Ophthalmology, University of CA – Los Angeles, David Geffen School of Medicine, Los Angeles, CA USA; 3 Jules Stein Eye Institute, University of CA – Los Angeles, Los Angeles, CA USA; 4 Department of Ophthalmology, Department of Health Services, Harbor-UCLA Medical Center, Los Angeles County, CA USA; 5 Tianjin Medical University Eye Hospital, Tianjin, China; 6 The Second Hospital of Tianjin Medical University, Tianjin, China; 7 Section of Ophthalmology, Department of Surgery, Cumming School of Medicine, University of Calgary, Calgary, Alberta Canada; 8 Vitreous Retina Macula Specialists of Toronto, Toranto, Ontario Canada; 9 Narayana Nethralaya Eye Institute, Bangalore, India

**Keywords:** Diabetic retinopathy, ETDRS, ICDR, Protocol AA, NPDR, PDR

## Abstract

**Purpose:**

To assess the relationship between qualitative diabetic retinopathy (DR) scales with the precise numbers and surface area of DR lesions within the Early Treatment Diabetic Retinopathy Study (ETDRS) standard seven field (S7F) region on ultrawide-field (UWF) color fundus images.

**Methods:**

In this study, we collected UWF images from adult patients with diabetes. Poor-quality images and eyes with any pathology precluding assessment of DR severity were excluded. The DR lesions were manually segmented. DR severity was graded according to the International Clinical Diabetic Retinopathy (ICDR) and AA protocol by two masked graders within the ETDRS S7F. These lesions’ numbers and surface area were computed and correlated against the DR scores using the Kruskal–Wallis H test. Cohen’s Kappa was performed to determine the agreement between two graders.

**Results:**

One thousand five hundred and twenty eyes of 869 patients (294 females, 756 right eyes) with a mean age of 58.7 years were included. 47.4% were graded as no DR, 2.2% as mild non-proliferative DR (NPDR), 24.0% as moderate NPDR, 6.3% as severe NPDR, and 20.1% as proliferative DR (PDR). The area and number of DR lesions generally increased as the ICDR level increased up to severe NPDR, but decreased from severe NPDR to PDR. There was perfect intergrader agreement on the DR severity.

**Conclusion:**

A quantitative approach reveals that DR lesions’ number and area generally correlate with ICDR-based categorical DR severity levels with an increasing trend in the number and area of DR lesions from mild to severe NPDR and a decrease from severe NPDR to PDR.

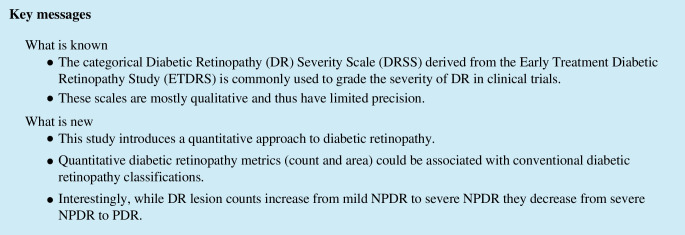

## Introduction

Diabetic retinopathy (DR) is one of the leading causes of blindness and visual impairment worldwide. It is predicted that the number of patients with diabetes will increase to roughly 642 million people by 2040 [1]. Due to advances in the systemic management of diabetes, patients have a longer life expectancy [2], and consequently, the prevalence of DR and vision-threatening DR (VTDR) are rising [3]. However, the percentage of VTDR (10.2%) is much lower than the overall prevalence of DR (34.6%) [3]; therefore, approaches for earlier detection of DR progression through screening assessments has been shown to played a significant role in the timely management of DR and prevention of its blinding complications [4].

Fundus photography has been among the frontline methods for DR screening [5]. Historically, mydriatic Early Treatment Diabetic Retinopathy Study (ETDRS) seven standard field (7SF) color fundus photographs have been the preferred approach for fundus imaging in diabetic patients. Compared to such conventional protocols, ultrawide-field (UWF) color imaging (UWF-CI) is more efficient because it provides a 200° field of view in a single capture and may also be acquired without mydriasis [6], which may yield better patient compliance. Previous studies have demonstrated a high level of agreement between ETDRS-7SF imaging and UWF-CI [7, 8]. Therefore, UWF-CI can be an accessible and practical modality for routine screening [9, 10], and potentially even for telemedicine programs [11].

The level of DR in fundus imaging was originally classified according to the Airlie House Classification of Diabetic Retinopathy [12]. The ETDRS modified this classification and characterized DR lesions more precisely with respect to their locations within the ETDRS-7SF [13]. The ETDRS system, however, was thought to be too complex for use in clinical practice, and thus the simplified International Clinical Diabetic Retinopathy (ICDR) Severity Scale (DRSS) [14] was developed. More recently, DR classification systems have been further modified to account for the peripheral fields beyond the seven ETDRS fields, which are now accessible as a result of UWF imaging. The best established is the methodology implemented in the Diabetic Retinopathy Clinical Research Network (DRCR.net) Protocol AA. Protocol AA also differentiated between eyes with predominantly peripheral lesions (PPL; i.e., more extensive DR lesions in any one peripheral field compared to its corresponding ETDRS field) and predominantly central lesions (PCL; each ETDRS field 3–7 has more extensive lesions compared to its corresponding peripheral field).

While DR classification systems have clearly evolved over time, these methods are largely qualitative. With recent advances in artificial intelligence and lesion segmentation, there may be opportunities to evaluate and score the disease in a more quantitative fashion. Sears et al. [15] measured the frequency and area of a variety of lesions (e.g., microaneurysm, hemorrhages) on UWF color images and demonstrated that quantitative classification of PCL and PPL could significantly differ from qualitative assessments. Sadda et al. [16] further highlighted that a quantitative approach in which all DR lesions were segmented could allow the location of each lesion relative to a landmark (e.g., optic nerve head or foveal center) to be described as distance measurement, thus potentially offering a more precise description of lesion distribution than the binary PCL versus PPL classification.

In this study, we further extend this quantitative approach to DR classification by correlating these quantitative DR lesion metrics with conventional DR severity levels.

## Methods

This was a multicenter retrospective cross-sectional study. UWF pseudocolor images were collected from patients with diabetes mellitus type 1 and 2 who were referred to Sankara Nethralaya (India), Narayana Nethralaya Eye Institute (India), Tianjin Medical University Eye Hospital (China), The Second Hospital of Tianjin (China), Vitreous Retina Macula Specialists of Toronto (Canada), and the Harbor UCLA centers and who had undergone UWF imaging. Images with evidence of retinal disease other than DR, such as retinal vascular occlusion (RVO), and ungradable images (any artifact or abnormality that precluded the assessment of DR lesions within the ETDRS 7SF) were excluded. Since it was a retrospective study, the medical records of the patients were not available for evaluation. The research was approved by the Institutional Review Board (IRB) of UCLA, but as the analysis was retrospective, a waiver of informed consent was granted. The research was consistent with the tenets set forth in the Declaration of Helsinki.

### Image acquisition

Nonmydriatic stereoscopic 200° UWF pseudocolor images were acquired using the Optos Daytona (Optos Plc, Dunfermline, Scotoland, United Kingdom) in India and China and the Optos P200Tx (Optos Plc) in Los Angeles and Canada. UWF images were deidentified, exported, and sent to the Doheny Image Reading and Research Laboratory for analysis.

### Image segmentation

**Fig. 1:**
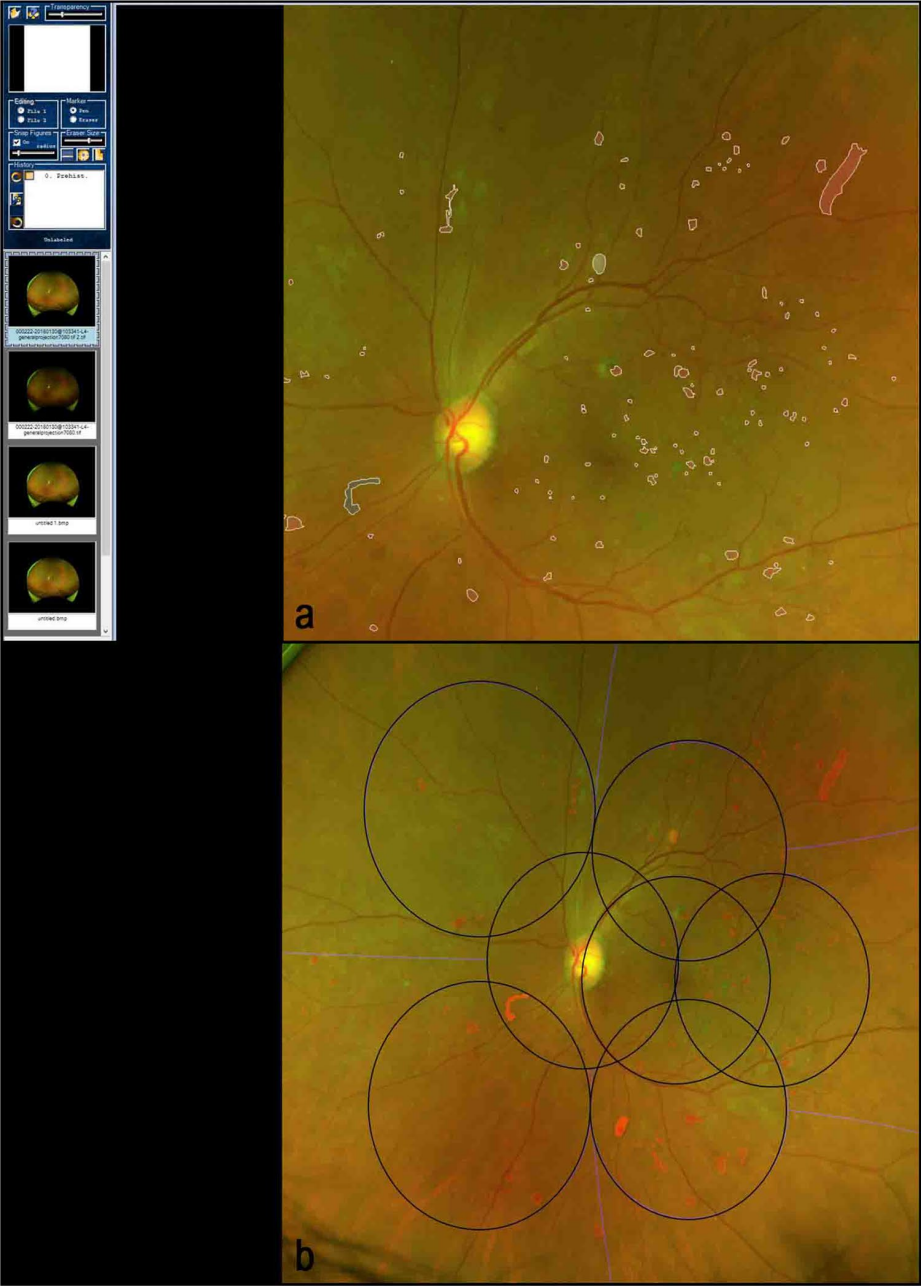
**a**: GRADOR software; demonstrating the segmentation of the diabetic retinopathy (DR) lesions in an eye with severe non-proliferative diabetic retinopathy (NPDR) according to the International Clinical DR (ICDR) and moderate NPDR according to the DR Clinical Research Network (DRCR). The boundaries of lesions are first outlined and then filled with a particular pen color to designate each type of lesion. **b**: The ultra-wide field color fundus image with overlaid Early Treatment Diabetic Retinopathy Study (ETDRS) seven standard fields. Within the ETDRS seven standard fields in this particular case, the total number and area of hemorrhages were 78.9 and 4.1, of microaneurysms were 103.5 and 1.1, of cotton wool spots were 1.0 and 0.2, and of IRMAs were 3.0 and 0.9. There was no evidence of venous loop, venous beading, exudate, preretinal hemorrhage, NVE, and NVD in this case

The images were automatically converted to TIF format using ImageJ (version 2.3.0/1.53f, developed by Wayne Rasband, National Institutes of Health, Bethesda, MD; available at https://rsb.info.nih.gov/ij/index.html). Similar to the approach described by Sears et al., the DR lesions in the entire retina were manually segmented using previously described planimetric GRADOR software, allowing users to load, pan, and zoom images and outline multiple features on the images using the mouse. Each individual image was graded by two graders in two rounds. In the first round, DR lesions were exhaustively segmented by one of the trained DR graders (YH, HE, HL, SFA, RG, DO). In the second round, all the images were then re-reviewed by one grader (HE) to inspect for and correct any subtle segmentation errors. The following DR lesions were segmented: intraretinal hemorrhages (H), microaneurysms (MA), hard exudates (HE), cotton wool spots (CWS), intraretinal microvascular abnormalities (IRMA), venous abnormalities, including venous loops (VL) and venous beading (VB), neovascularization of the disc (NVD), neovascularization elsewhere (NVE), preretinal/vitreous hemorrhages (PRH/VH), fibrous proliferation of the disc (PFD) and fibrous proliferation elsewhere (PFE) (Fig. 1-A). For structures with poorly defined boundaries, such as venous beading, the grader attempted to define the zone of involvement of the vessel as accurately as possible. For such lesions, the presence and frequency of the findings were deemed to be meaningful, but the area was not.

### Image analysis

The geometric axes of the optic nerve head and foveal centers were defined using ImageJ version 2.3.0/1.53f. The geometric axes were used to define the position of the ETDRS fields on UWF images in the following steps. The images were renamed according to these axes and the eye laterality. The segmented images were also stereographically projected before processing, as explained in previous publications [15]. Then, the projected renamed images were uploaded to an image processing tool (Optos Plc) which computed the number and surface area of every individual DR lesion and also applied a mask corresponding to the region of the ETDRS 7SF onto the UWF-C images (Fig. 1-B). The frequency for a particular case may not be integers because the lesion may straddle two different fields and may be only partially counted in one field.

### Image grading

The segmented images that were overlayed with the ETDRS 7SF mask were then graded according to both DRCR protocol AA and ICDR by the same trained graders (YH, HL, SFA, DO, RG, YA) who annotated the images. These images were all graded by a second grader (HE) masked to the first graders' scores. Protocol AA consists of 17 levels ranging from level 10 "DR absent" to level 90 "cannot grade" (Table 1); fields 2–7 were assessed per the ETDRS report 10 protocol [13], and images were compared with the reference standard ETDRS images. Any significant pathology interfering with DR lesion assessment was exclusionary, so images with levels 81 to 90 were excluded from subsequent analysis. The ICDR classification consists of five stages ranging from level 0 "No DR" to 4 "PDR," as shown in Table 1.

**Table 1:** Classifications of diabetic retinopathy (DR) based on the International Clinical Diabetic Retinopathy (ICDR) and Diabetic Retinopathy Clinical Research Network (DRCR) protocol AA scales. (N)PDR: (Non)proliferative DR

Classification system	Severity level	Score
ICDR	No DR	0
	Mild NPDR	1
	Moderate NPDR	2
	Severe NPDR	3
	PDR	4
DRCR protocol AA	DR absent	10
	Non-DR abnormalities	12
	DR questionable	14
	DR questionable	15
	Ma only	20
	Mild NPDR	35
	Moderate NPDR	43
	Moderately Severe NPDR	47
	Severe NPDR	53
	Very Severe NPDR	53E
	Inactive PDR	60
	Mild PDR	61
	Moderate PDR	65
	High-risk PDR	71/75
	Cannot grade	81/85/90

### Outcome measures and statistics

The total frequency and surface area of every type of DR lesion within the ETDRS 7SF were calculated. A Kruskal–Wallis H test and subsequent Dunn's procedure with a Bonferroni correction were conducted using SPSS (IBM Corp. Released 2019. IBM SPSS Statistics for Windows, Version 26.0. Armonk, NY: IBM Corp) and RStudio (2022.02.3–492, R version 4–2-1, Posit, Boston, USA) to determine if the parameters were significantly different between DR severity levels. First, the differences were evaluated between different ICDR levels (mild NPDR, moderate NPDR, severe NPDR, PDR), and then the differences were assessed for subclasses of NPDR and PDR according to protocol AA. Descriptive measures were presented as median and mean ± standard deviation (SD). The frequency and area of PFD and PFE were not reported since these metrics did not impact DR severity. Cohen's Kappa was run to determine the agreement between the two graders. A p-value less than 0.05 was considered significant.

## Results

One-thousand six-hundred fifty-one eyes were assessed, from which 106 eyes were excluded due to poor image quality, 13 eyes were excluded due to the presence of RVO, and 12 eyes were excluded due to pathologies interfering with DR assessment within the area of ETDRS 7SF (e.g., asteroid hyalosis, DR severity levels 81 to 90). Thus, 1520 eyes (765 right, 755 left) of 869 patients (294 female, 575 male) with a mean age of 58.7 (± 12.5; range 23–114) were included in the final analysis. Of these eyes, 721 (47.4%) were diagnosed as no DR, 33 (2.2%) as mild NPDR, 365 (24.0%) as moderate NPDR, 95 (6.3%) as severe NPDR, and 306 (20.1%) as PDR based on ICDR (Table 2). Patients with questionable DR were included in the mild NPDR subgroup. The number and percentage of eyes within each group according to the protocol AA classification are also summarized in Table 2. There was perfect agreement between the two graders (κ = 0.881, *p* < 0.05) [17].

**Table 2:** Mean, median, and total frequency and area of diabetic retinopathy (DR) lesions within the Early Treatment Diabetic Retinopathy Study seven standard fields according to International Clinical Diabetic Retinopathy (ICDR) and DRCR protocol AA scales

	DR lesions	Frequency/ area (mm)^2^	ICDR				DRCR protocol			
			Non-proliferative DR			Proliferative DR	Questionable DR	Microaneurysm(s) only	Non-proliferative DR	
			Mild	Moderate	Severe				Mild	Moderate
Mean (SD) [median]	Venous loop	frequency	0.00 (0.00) [0.00]	0.01 (0.09) [0.00]	0.04 (0.25) [0.00]	0.19 (0.66) [0.00]	0.00 (0.00) [0.00]	0.00 (0.00) [0.00]	0.08 (0.01) [0.00]	0.13 (0.02) [0.00]
		area	0.00 (0.00) [0.00]	0.01 (0.02) [0.00]	0.01 (0.05) [0.00]	0.03 (0.15) [0.00]	0.00 (0.00) [0.00]	0.00 (0.00) [0.00]	0.02 (0.01) [0.00]	0.02 (0.01) [0.00]
	Hemorrhage	frequency	0.00 (0.00) [0.00]	26.47 (27.57) [20.18]	78.67 (65.23) [108.94]	40.96 (40.97) [29.67]	6.23 (1.73) [1.00]	0.00 (0.00) [0.00]	21.62 (1.19) [16.70]	39.35 (5.04) [58.59]
		area	0.00 (0.00) [0.00]	1.43 (1.64) [1.02]	5.45 (5.15) [8.35]	3.11 (5.75) [1.76]	0.31 (0.09) [0.06]	0.00 (0.00) [0.00]	1.11 (0.07) [0.81]	2.84 (0.37) [3.72]
	Microaneurysm	frequency	6.33 (6.51) [4.50]	20.12 (21.90) [13.49]	40.86 (36.30) [37.40]	27.52 (26.76) [20.00]	0.97 (0.27) [0.00]	6.51 (1.46) [4.50]	19.30 (1.06) [11.00]	28.24 (3.62) [29.06]
		area	0.06 (0.06) [0.04]	0.17 (0.22) [0.11]	0.36 (0.42) [0.28]	0.22 (0.27) [0.13]	0.86 (0.24) [0.00]	0.07 (0.02) [0.04]	0.20 (0.02) [0.08]	0.31 (0.04) [0.28]
	Hard exudate	frequency	0.00 (0.00) [0.00]	39.55 (87.89) [6.96]	65.80 (91.01) [37.20]	48.64 (99.46) [9.32]	32.47 (9.01) [6.00]	0.00 (0.00) [0.00]	90.73 (4.96) [6.0]0	49.61 (6.36) [12.83]
		area	0.00 (0.00) [0.00]	1.79 (6.03) [0.19]	1.79 (2.83) [1.04]	1.94 (5.28) [0.30]	0.27 (0.08) [0.13]	0.00 (0.00) [0.00]	6.25 (0.35) [0.16]	3.12 (0.40) [0.31]
	Cotton wool spot	frequency	0.00 (0.00) [0.00]	2.21 (4.72) [0.00]	3.74 (6.21) [0.00]	1.23 (3.51) [0.00]	1.27 (0.36) [0.00]	0.00 (0.00) [0.00]	4.66 (0.26) [0.00]	5.00 (0.64) [0.00]
		area	0.00 (0.00) [0.00]	0.59 (1.30) [0.00]	0.84 (1.43) [0.00]	0.85 (8.62) [0.00]	5.26 (1.46) [0.00]	0.00 (0.00) [0.00]	1.27 (0.07) [0.00]	1.57 (0.21) [0.00]
	intraretinal microvascular abnormalities (IRMA)	frequency	0.00 (0.00) [0.00]	0 (0) [0.00]	0.82 (1.42) [0.00]	0.63 (1.51) [0.00]	0.00 (0.00) [0.00]	0.00 (0.00) [0.00]	0.00 (0.00) [0.00]	1.26 (0.17) [0.00]
		area	0.00 (0.00) [0.00]	0.01 (0.01) [0.00]	0.19 (0.61) [0.00]	0.23 (1.17) [0.00]	0.26 (0.08) [0.00]	0.00 (0.00) [0.00]	0.01 (0.01) [0.00]	0.26 (0.04) [0.00]
	Pre-retinal/Vitreous hemorrhage	frequency	0.00 (0.00) [0.00]	0.00 (0.00) [0.00]	0.00 (0.00) [0.00]	0.34 (1.40) [0.00]	0.00 (0.00) [0.00]	0.00 (0.00) [0.00]	0.00 (0.00) [0.00]	0.00 (0.00) [0.00]
		area	0.00 (0.00) [0.00]	0.00 (0.00) [0.00]	0.00 (0.00) [0.00]	1.79 (6.95) [0.00]	0.00 (0.00) [0.00]	0.00 (0.00) [0.00]	0.00 (0.00) [0.00]	0.00 (0.00) [0.00]
	Neovascularization elsewhere	frequency	0.00 (0.00)	0.00 (0.00)	0.00 (0.00)	1.79 (6.95) [0.00]	0.00 (0.00) [0.00]	0.00 (0.00) [0.00]	0.00 (0.00) [0.00]	0.00 (0.00)
		area	0.00 (0.00)	0.00 (0.00)	0.00 (0.00)	3.28 (11.33) [0.00]	0.00 (0.00) [0.00]	0.00 (0.00) [0.00]	0.00 (0.00)	0.00 (0.00)
	Venous beading	frequency	0.00 (0.00)	0.00 (0.00)	0.29 (0.66) [0.00]	0.23 (0.72) [0.00]	1.38 (0.39) [0.00]	0.00 (0.00) [0.00]	0.00 (0.00)	0.00 (0.00)
		area	0.00 (0.00) [0.00]	0.00 (0.00) [0.00]	0.19 (0.56) [0.00]	0.15 (0.55) [0.00]	0.99 (0.28) [0.00]	0.00 (0.00) [0.00]	0.00 (0.00)	0.00 (0.00)
	Neovascularization of the disc	frequency	0.00 (0.00) [0.00]	0.00 (0.00) [0.00]	0.00 (0.00) [0.00]	0.35 (0.75) [0.00]	0.00 (0.00) [0.00]	0.00 (0.00) [0.00]	0.00 (0.00)	0.00 (0.00)
		area	0.00 (0.00) [0.00]	0.00 (0.00) [0.00]	0.00 (0.00) [0.00]	6.05 (25.73) [0.00]	0.00 (0.00) [0.00]	0.00 (0.00) [0.00]	[0.00]	[0.00]
Number (percent%) of Eyes			33 (2.2%)	365 (24%)	95 (6.3%)	306 (20.1%)	13 (0.9%)	20 (1.3%)	335 (22%)	61 (4%)

	DR lesions	Frequency/ area (mm)^2^	DRCR protocol							Total
			Non-proliferative DR			Proliferative DR				
			Moderately severe	Severe	Very Severe	Inactive	Mild	Moderate	High risk	
Mean (SD) [median]	Venous loop	frequency	0.27 (0.05) [0.00]	0.37 (0.07) [0.00]	0.00 (0.00) [0.00]	0.56 (0.05) [0.00]	0.55 (0.09) [0.00]	0.70 (0.10) [0.00]	0.84 (0.10) [0.00]	.042 (0.32) [0.00]
		area	0.07 (0.02) [0.00]	0.06 (0.02) [0.00]	0.00 (0.00) [0.00]	0.15 (0.02) [0.00]	0.07 (0.01) [0.00]	0.07 (0.01) [0.00]	0.23 (0.03) [0.00]	0.01 (0.07) [0.00]
	Hemorrhage	frequency	43.99 (8.32) [44.54]	66.49 (12.14) [119.85]	69.99 (28.58) [122.37]	35.67 (3.19) [19.86]	41.84 (6.46) [32.40]	46.21 (6.12) [36.19]	43.26 (4.78) [36.66]	19.69 (36.09) [0.00]
		area	3.11 (0.59) [2.17]	5.14 (0.94) [8.83]	6.32 (2.58) [9.73]	2.66 (0.24) [1.15]	2.65 (0.41) [1.92]	3.25 (0.43) [1.99]	9.82 (1.09) [3.15]	1.32 (3.40) [0.00]
	Microaneurysm	frequency	36.58 (6.92) [21.99]	38.81 (7.09) [55.27]	44.95 (18.35) [43.09]	21.44 (1.92) [16.00]	39.59 (6.11) [30.82]	24.36 (3.23) [23.00]	25.55 (2.83) [20.73]	13.12 (23.00) [0.00]
		area	0.42 (0.08) [0.16]	0.52 (0.10) [0.42]	0.48 (0.20) [0.51]	0.22 (0.02) [0.09]	0.43 (0.07) [0.22]	0.26 (0.04) [0.12]	0.24 (0.03) [0.16]	0.11 (0.23) [0.00]
	Hard exudate	frequency	120.75 (22.82) [19.70]	90.67 (16.56) [37.62]	81.44 (33.25) [55.72]	119.23 (10.67) [8.00]	110.05 (16.99) [21.08]	86.06 (11.40) [7.00]	62.17 (6.87) [9.28]	23.60 (70.41) [0.00]
		area	3.09 (0.59) [0.48]	2.17 (0.40) [1.10]	1.69 (0.69) [1.18]	7.33 (0.66) [0.22]	3.10 (0.48) [0.64]	2.85 (0.38) [0.34]	3.44 (0.38) [0.37]	0.94 (3.98) [0.00]
	Cotton wool spot	frequency	5.94 (1.13) [2.00]	7.33 (1.34) [0.00]	7.14 (2.92) [5.62]	2.28 (0.21) [0.00]	3.39 (0.53) [0.00]	6.16 (0.82) [0.00]	2.38 (0.27) [0.01]	1.02 (3.41) [0.00]
		area	1.06 (0.20) [0.37]	1.68 (0.31) [0.00]	1.40 (0.57) [1.14]	0.72 (0.07) [0.00]	23.14 (3.57) [0.00]	1.01 (0.14) [0.00]	1.03 (0.12) [0.01]	0.37 (3.96) [0.00]
	intraretinal microvascular abnormalities (IRMA)	frequency	0.19 (0.04) [0.00]	1.50 (0.28) [0.00]	2.41 (0.99) [0.00]	1.19 (0.11) [0.00]	2.29 (0.36) [0.00]	1.73 (0.23) [0.00]	1.23 (0.14) [0.00]	0.18 (0.82) [0.00]
		area	0.09 (0.02) [0.00]	0.14 (0.03) [0.00]	2.22 (0.91) [0.00]	0.23 (0.03) [0.00]	0.91 (0.14) [0.00]	0.89 (0.12) [0.00]	2.01 (0.23) [0.00]	0.06 (0.56) [0.00]
	Pre-retinal/Vitreous hemorrhage	frequency	0.00 (0.00) [0.00]	0.00 (0.00) [0.00]	0.00 (0.00)	0.00 (0.00)	0.00 (0.00) [0.00]	0.31 (0.05) [0.00]	2.50 (0.28) [0.00]	0.07 (0.64) [0.00]
		area	0.00 (0.00) [0.00]	0.00 (0.00) [0.00]	0.00 (0.00) [0.00]	0.00 (0.00) [0.00]	0.00 (0.00) [0.00]	1.42 (0.19) [0.00]	12.21 (1.35) [0.00]	0.36 (3.21) [0.00]
	Neovascularization elsewhere	frequency	0.00 (0.00)	0.00 (0.00)	0.00 (0.00)	0.00 (0.00)	1.92 (0.30) [1.16]	2.05 (0.28) [1.78]	1.96 (0.22) [1.01]	0.22 (0.91) [0.00]
		area	0.00 (0.00)	0.00 (0.00)	0.00 (0.00)	0.00 (0.00)	1.05 (0.17) [0.70]	9.48 (1.26) [3.51]	19.47 (2.15) [1.69]	0.67 (5.27) [0.00]
	Venous beading	frequency	0.63 (0.12) [0.00]	0.67 (0.13) [0.00]	1.38 (0.57) [0.90]	0.49 (0.05) [0.00]	1.04 (0.16) [0.00]	0.68 (0.10) [0.00]	0.82 (0.10) [0.00]	0.07 (0.38) [0.00]
		area	0.61 (0.12) [0.00]	0.40 (0.08) [0.00]	1.46 (0.60) [0.21]	0.25 (0.03) [0.00]	0.94 (0.15) [0.00]	0.34 (0.05) [0.00]	0.72 (0.08) [0.00]	0.04 (0.30) [0.00]
	Neovascularization of the disc	frequency	0.00 (0.00)	0.00 (0.00)	0.00 (0.00)	0.00 (0.00)	0.00 (0.00)	0.72 (0.10) [0.00]	1.01 (0.12) [1.13]	0.07 (0.36) [0.00]
		area	[0.00]	[0.00]	[0.00]	[0.00]	[0.00]	3.78 (0.51) [0.00]	46.14 (5.10) [2.58]	1.23 (11.83) [0.00]
Number (percent%) of Eyes			28 (1.8%)	30 (2%)	6 (0.4%)	125 (8.2%)	42 (2.8%)	57 (3.8%)	82 (5.4%)	1520 (100%)

### Frequency and surface area

Total, median, and mean values for the frequency and area of each lesion within the ETDRS 7SF according to DR severity levels based on ICDR and protocol AA classifications are shown in Table 2. In addition, the precise frequency and surface area of DR lesions were recorded for the case illustrated in Fig. 1.

### Comparison of DR lesions metrics within DR severity levels

**Fig. 2:**
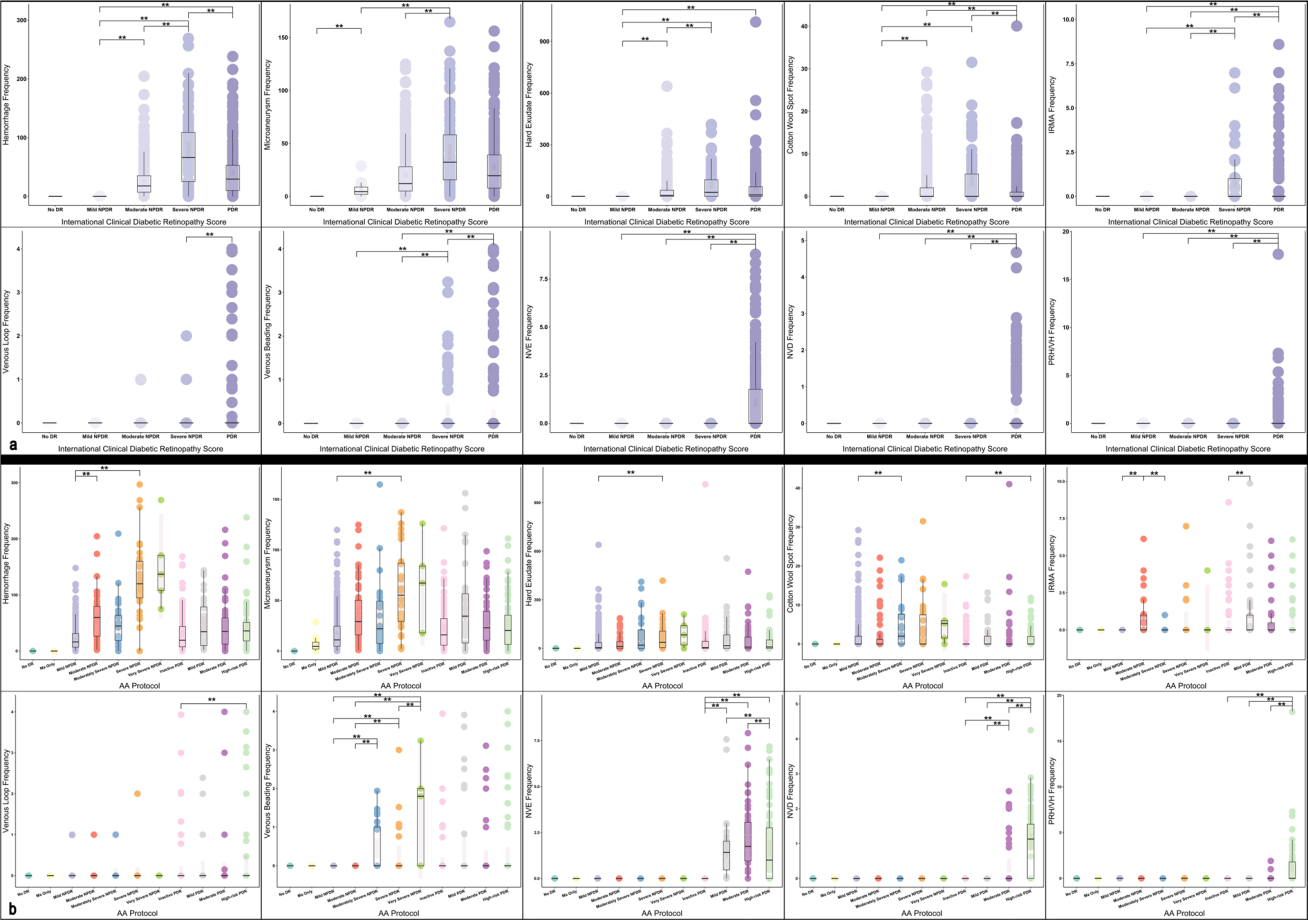
Box and whisker plots demonstrating the frequency of diabetic retinopathy lesions within the Early Treatment Diabetic Retinopathy Study (ETDRS) seven standard fields according to the International Clinical Diabetic Retinopathy (ICDR) (**a**) and the Diabetic Retinopathy Clinical Research Network (DRCR) protocol AA (**b**). Statically significant comparisons are shown with the bars above the plots. The pairwise comparisons with the "No DR" level have not been shown since this level was significant with most of the others. Grey rectangle: confidence interval

**Fig. 3:**
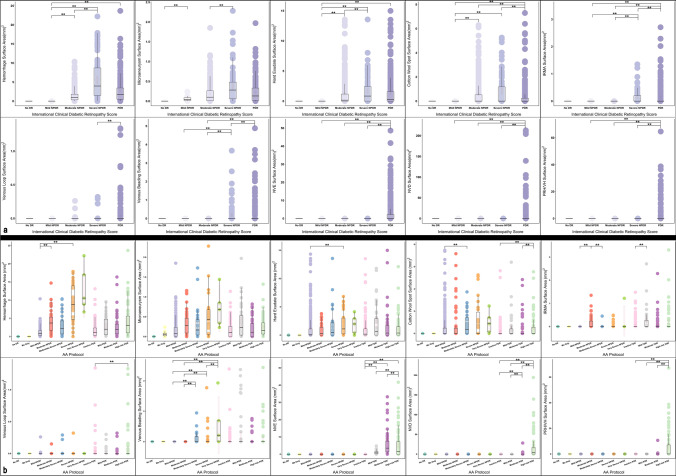
Box and whisker plots demonstrating the surface area (mm^2^) of diabetic retinopathy lesions within the Early Treatment Diabetic Retinopathy Study (ETDRS) seven standard fields according to the International Clinical Diabetic Retinopathy (ICDR) (**a**) and the Diabetic Retinopathy Clinical Research Network (DRCR) protocol AA (**b**). Statically significant comparisons are shown with the bars above the plots. The pairwise comparisons with the "No DR" level have not been shown since this level was significant with most of the others. Grey rectangle: confidence interval, mm^2^: square millimeter

A Kruskal–Wallis H test showed statistically significant differences between the median number of DR lesions and the DR lesion surface area (Table 3). Pairwise comparisons within ICDR and protocol AA levels are shown in Figs. 2 and 3.

**Table 3:** Results of the Kruskal–Wallis H test showing the statistically significant differences in the frequency and area of DR lesions between levels of DR severity classifications (International Clinical Diabetic Retinopathy (ICDR) and protocol AA)

Type of lesion	Frequency/ area (mm)	International clinical diabetic retinopathy		Protocol AA	Degree of freedom	*P* value
		H value	Degree of freedom	H value		
Venous loop	frequency	114.07	11	121.78	13	0.00
	area	113.97	11	121.68	13	0.00
Hemorrhage	frequency	1281.83	11	1364.26	13	0.00
	area	1285.78	11	1354.67	13	0.00
Microaneurysm	frequency	1224.18	11	1310.20	13	0.00
	area	950.23	11	1008.57	13	0.00
Hard exudate	frequency	654.50	11	692.32	13	0.00
	area	653.23	11	688.58	13	0.00
Cotton wool spot	frequency	390.06	11	411.85	13	0.00
	area	391.07	11	412.41	13	0.00
Intraretinal microvascular abnormalities (IRMA)	frequency	368.55	11	395.38	13	0.00
	area	359.31	11	384.86	13	0.00
Preretinal/Vitreous hemorrhage	frequency	562.96	11	621.62	13	0.00
	area	563.24	11	623.25	13	0.00
Neovascularization elsewhere	frequency	1047.11	11	1093.20	13	0.00
	area	1045.13	11	1089.86	13	0.00
Venous beading	frequency	248.86	11	269.77	13	0.00
	area	248.82	11	269.85	13	0.00
Neovascularization of the disc	frequency	809.44	11	866.47	13	0.00
	area	815.27	11	872.20	13	0.00

The median frequency and area of MA, H, and HE demonstrated the same trend. Specifically, the lesion counts increased from "No DR" to the severe NPDR level and then decreased in eyes with PDR. The pairwise comparisons for frequencies were statistically significant for H and HE for all DR severity levels (*p* < 0.05, χ2(3) = 1281.834 and 654.501, respectively) but were not significant for MA (χ2(3) = 1194.858) (Fig. 2). The pairwise comparisons of median area for H and HE were overall statistically significant (*p* < 0.05, χ2(3) = 1285.776 and 654.501, respectively) but were not significant for MA (χ2(3) = 921.817) (Fig. 3).

The frequency and area of IRMA increased significantly from severe NPDR to PDR (*p* < 0.05, χ2(3) = 339.905 and 334.942, respectively) (Figs. 2 and 3). The median number of CWS increased from moderate NPDR to severe NPDR (*p* = 0.07), but then were lower at the PDR level compared to moderate NPDR (*p* < 0.05, χ2(3) = 333.181) (Fig. 2). The area of CWS increased from mild to moderate NPDR (*p* < 0.05) and slightly increased from moderate NPDR to PDR (*p* = 0.12, χ2(3) = 332.304) (Fig. 3).

The number and area of VL were highest at the PDR level (*p* < 0.05, χ2(3) = 94.629 and 94.412, respectively) (Figs. 2 and 3). The number and area of VB increased significantly from moderate to severe NPDR (*p* < 0.05) (Figs. 2 and 3). From severe NPDR to PDR, however, the number and area of VB decreased (*p* = 0.07, χ2(3) = 126.686 and 126.556, respectively) (Figs. 2 and 3).

Within different sublevels of the PDR stage, the number and area of NVD significantly increased from mild PDR to high-risk PDR (*p* < 0.05, χ2(3) = 809.441 and 815.273, respectively) (Figs. 2 and 3). The number and area of NVEs also increased from mild to moderate PDR (*p* = 1) but then decreased from moderate to high-risk PDR (*p* < 0.05, χ2(3) = 1047.111 and 1045.129, respectively) (Figs. 2 and 3). The median number and area of the PRH/VH were highest in high-risk PDR (*p* < 0.05, χ2(3) = 562.958 and 563.239, respectively) (Figs. 2 and 3).

## Discussion

In this study of 1520 eyes of 869 diabetic patients, we precisely quantified the number and surface area of various DR lesions (VL, MA, H, HE, CWS, IRMA, NVE, NVD, VB, and PRH/VH) within the ETDRS 7SF region on UWF-C images and assessed the correlation between these quantitative metrics with standard ICDR and protocol AA classifications of overall DR severity. We observed that while the frequency of most DR lesions increased with more advanced DR severity levels, many lesions, such as H and MA, decreased from severe NPDR to PDR.

MAs are perhaps the most extensively studied DR lesions to date, and it has been shown that their frequency is a risk factor for DR progression [18–21]. These studies have primarily evaluated alterations in the number of MAs over time. Our study was cross-sectional and demonstrated that while the number of MA increased with the severity of NPDR, there is an apparent trend for a decrease at the PDR level (Fig. 2). This observation is consistent with Sun et al. [22], who observed that the number of MAs both in the entire retina and in retinal subregions on UWF fluorescein angiography (UWF-FA) images showed the same trend between ICDR levels. Kohner et al. [18] showed that the number of MA in the fluorescein angiogram primarily increased, but over time, their number reflected a declining slope. Kohner et al. [23] also demonstrated the tendency of MA to disappear over time, particularly when fewer MA were present when DR was first detected. Santos et al. [24] also observed a falling trend in the MA count in color fundus images over a five-year follow-up period; however, the disappearance of MA in their study occurred while DR progressed from mild NPDR to moderate NPDR (*p* < 0.05), which stands in contrast to our study, where the decline in numbers was observed between severe NPDR and PDR.

In contrast to these studies, Ehlers et al. [25] observed a positive correlation between pan-retinal MA frequency and DR severity in UWF-FA and associated color images. Ehlers et al., however, considered the entire UWF region, as opposed to the ETDRS-7SF region assessed in our study, which was focused on correlation with existing severity scales. Given that it has been shown that MAs may disappear from one location and appear in another over time [24] and are more numerous in the central retina [26], it is possible that they were disappearing centrally and becoming more manifest peripherally as the eyes progressed from severe NPDR to PDR.

The pathophysiology driving microaneurysm turnover and the apparent central disappearance of MA in more advanced (i.e., PDR) stages of DR remains incompletely defined. We might speculate, however, that as the extent of non-perfusion increases as DR progresses [27–30], the loss of capillaries will be associated with the disappearance of the microaneurysms associated with these capillaries. Further longitudinal, quantitative UWF-FA based studies will be required, however, to better evaluate this issue.

A similar pattern with regard to DR lesion extent and DR severity was observed for the frequency and area of H and HE and the frequency of IRMA. All demonstrated an increase from no DR to severe NPDR followed by a decrease from severe NPDR to PDR (*p* < 0.05). In contrast to this observation, Sadda et al. [16] noted a continuous increase in the number of hemorrhages with a DR severity level. Kohner et al. [18] demonstrated that the number of H and HE steadily increased over a two-year follow-up, but the number of IRMA initially increased with a subsequent decrease over the same period of time. As far as we know, no other study has evaluated these quantitative metrics against DR levels. The reason for the inconsistency among studies is uncertain but may be related to differences in the patient populations – for example, the present study is a multicenter study including several different ethnicities, and there are known differences in the appearance of DR among different ethnicities. Another explanation may be related to the limitations of a retrospective analysis with varying numbers of subjects at various severity levels. A possible explanation for why the number of H, HE, and IRMA decrease from severe NPDR to PDR may be similar to that for MA, in which progressive and chronic non-perfusion can eliminate MAs and telangiectatic capillaries that may be the source of these hemorrhages and exudates.

As IRMA tend to arise in regions with CWS [31], it is perhaps not surprising that CWS showed a similar trend as IRMA. Similar to the IRMA area, the CWS area increased with increasing DR severity level, though the differences between severe NPDR and PDR levels were not statically significant. Similarly, Kohner et al. [18] observed an initial upward and then a downward trend for the frequency of CWS over time.

In parallel with the other DR lesions, the number and area of VB increased from moderate to severe NPDR (*p* < 0.05) and then showed a decreasing trend from severe NPDR to PDR. However, the number and area of VL in the PDR group were significantly higher compared to the less severe levels (*p* < 0.05). Kohner et al. [18] did not find any correlation between venous abnormalities and DR severity, but considered these lesions as indicators of advanced DR stages. It is evident that the underlying pathology of venous abnormalities is ischemia, but they arise from veins instead of capillaries.

NVEs and NVDs occur due to the release of vascular endothelial growth factor (VEGF) from neighboring cells after prolonged ischemia [32, 33]. PRH/VHs can then occur as a result of vitreous traction on these fragile nascent vessels. As expected, in our study, the number and frequency of NVDs and PRH/VH increased from mild PDR to high-risk PDR, which is consistent with their definitions. Nevertheless, the number and area of NVEs increased from mild to moderate PDR and decreased in high-risk PDR (*p* < 0.05).

Our study has a number of limitations that should be considered when assessing our results. Most significantly, this was a retrospective study and was thus limited by ascertainment bias. In particular, whether a UWF image was available was dependent on the pattern of practice of the particular clinical center. It is possible and perhaps likely that the indications for obtaining UWF-CI images may have significantly varied among centers. Another consequence of the retrospective ascertainment was an uneven distribution of cases among DR severity levels. We had many subjects with moderate NPDR, but very few with mild NPDR. As a result, we were likely underpowered to identify small differences between some of these severity levels. Another significant limitation of our analysis is that we did not have access to the complete medical records of these subjects and thus could not adjust for potential confounders, such as glycemic control, the duration of diabetes, and other systemic diseases (e.g., hypertension, dyslipidemia). Furthermore, although we could inspect images for the presence of laser scars, we could not verify by our own inspection whether a patient may have received pharmacotherapy for their DR or diabetic macular edema. As DR lesions are known to regress with anti-VEGF therapy, this could impact the quantitative comparisons. It should be noted that none of the centers included in this study used anti-VEGF therapy for the treatment of non-proliferative retinopathy though they used this treatment for diabetic macular edema. Our study does have several strengths, including the use of certified DR graders, dual grading to assess repeatability, and an exhaustive manual segmentation-based quantitative approach. At the same time, we recognize that progress in deep-learning-based methods should allow these quantitative parameters to be generated automatically in the future.

In summary, despite the limitations, our study highlights that a quantitative approach to DR lesion assessment offers an opportunity to more precisely describe the phenotype of DR, which may provide new insights into the evolution of the disease. Further studies needed to validate this study for further clinical application and our findings will clearly require replication in a prospective longitudinal study, but perhaps most immediately, these approaches could be applied to the Protocol AA image dataset when it becomes publicly available for external analysis. Characterizing the natural evolution of DR at the lesion level may be of particular importance in this era of pharmacotherapeutics, which appear to have an impact on the underlying background retinopathy.
